# Decentralized colonoscopic surveillance with high patient compliance prevents hereditary and familial colorectal cancer

**DOI:** 10.1007/s10689-016-9867-7

**Published:** 2016-03-02

**Authors:** Olle Sjöström, Lars Lindholm, Björn Tavelin, Beatrice Melin

**Affiliations:** 1 Department of Radiation Sciences, Oncology, Unit of Research, Education and Development-Östersund, Umeå University, Umeå, Sweden; 2 Department of Public Health and Clinical Medicine, Epidemiology and Global Health, Umeå University, Umeå, Sweden; 3 Department of Radiation Sciences, Oncology, Umeå University, Umeå, Sweden

**Keywords:** Colorectal cancer, Surveillance colonoscopy, Cancer prevention, Hereditary colorectal

## Abstract

Although colonoscopic surveillance is recommended both for individuals with known hereditary colorectal cancer (HCRC) syndromes and those with a more moderate familial colorectal cancer (FCRC) history, the evidence for the benefits of surveillance is limited and surveillance practices vary. This study evaluates the preventive effect for individuals with a family history of CRC of decentralized colonoscopic surveillance with the guidance of a cancer prevention clinic. We performed a population based prospective study of 261 patients with HCRC or FCRC, recorded in the colonoscopic surveillance registry at the Cancer genetics clinic, University Hospital of Umeå, Sweden. Colonoscopic surveillance was conducted every second (HCRC) or fifth (FCRC) year at local hospitals in Northern Sweden. Main outcome measures were findings of high-risk adenomas (HRA) or CRC, and patient compliance to surveillance. Estimations of the expected numbers of CRC without surveillance were made. During a total of 1256 person years of follow-up, one case of CRC was found. The expected numbers of cancers in the absence of surveillance was between 9.5 and 10.5, resulting in a standardized incidence ratio, observed versus expected cases of CRC, between 0.10 (CI 95 % 0.0012–0.5299) and 0.11 (CI 95 % 0.0014–0.5857). No CRC mortality was reported, but three patients needed surgical intervention. HRA were found in 5.9 % (14/237) of the initial and in 3.4 % (12/356) of the follow-up colonoscopies. Patient compliance to the surveillance program was 90 % as 597 of the planned 662 colonoscopies were performed. The study concludes that colonoscopic surveillance with high patient compliance to the program is effective in preventing CRC when using a decentralized method for colonoscopy surveillance with the guidance of a cancer prevention clinic.

**Electronic supplementary material:**

The online version of this article (doi:10.1007/s10689-016-9867-7) contains supplementary material, which is available to authorized users.

## Introduction

A family history of colorectal cancer (CRC) is a well-known risk factor for developing cancer [1–4]. CRC risk increases according to the number of relatives diagnosed with CRC and the number of relatives diagnosed with early age CRC. Consequently, families with a strong dominant pattern of inheritance, indicating a hereditary colorectal cancer (HCRC) syndrome, have a higher risk compared to families with a more moderate clustering (i.e., familial colorectal cancer, FCRC).

In families with known HCRC syndromes, such as Lynch syndrome, colonoscopic surveillance may reduce colorectal cancer incidence and mortality [5]. Hence, surveillance in Lynch syndrome is well established and international guidelines recommend annual or biennial colonoscopies from the age of 20–25 [3, 6, 7]. For FCRC, however, the evidence of the benefits of colonoscopic surveillance is more limited [8–10]. As a consequence, international guidelines and practices for surveillance in FCRC are very divergent regarding when surveillance should start and the length of the examination intervals. The recommendations for starting surveillance ranges between 25 and 50 years old with intervals of 3–5 years [6, 7].

In the Northern Sweden Health Care Region, all individuals recommended for HCRC and FCRC surveillance since 1995 are prospectively recorded in a quality register at the Cancer Prevention Clinic at the University Hospital in Umeå, Sweden.

This study evaluates the CRC preventive effect for the individuals in the registry in order to optimize future strategies for surveillance. The evaluation includes analyzing the colonoscopic findings and describing patient compliance and the decentralized organization for the surveillance.

## Methods

### Material and procedures

The study subjects were prospectively recorded in the colonoscopic surveillance registry at the Cancer Prevention Clinic at Umeå University Hospital from 1995 to 1 September 2012. Colonoscopic surveillance was offered to individuals with an estimated lifetime risk of colorectal cancer of at least 10 % or in a few cases due to strong psychological preferences [3, 6, 7]. No individuals with previous CRC were included in the study.

The surveillance registry comprises data on age, sex, place of residence, estimated cancer risk, and when applicable microsatellite instability and immunohistochemistry or genetic screening for hereditary non-polyposis colorectal cancer (HNPCC/Lynch syndrome) genes. In addition were surveillance intervals and planned and performed surveillance colonoscopies recorded. The findings of the colonoscopies were documented including any incomplete examinations (inadequate bowel cleaning or failure to reach the caecum).

**Fig. 1:**
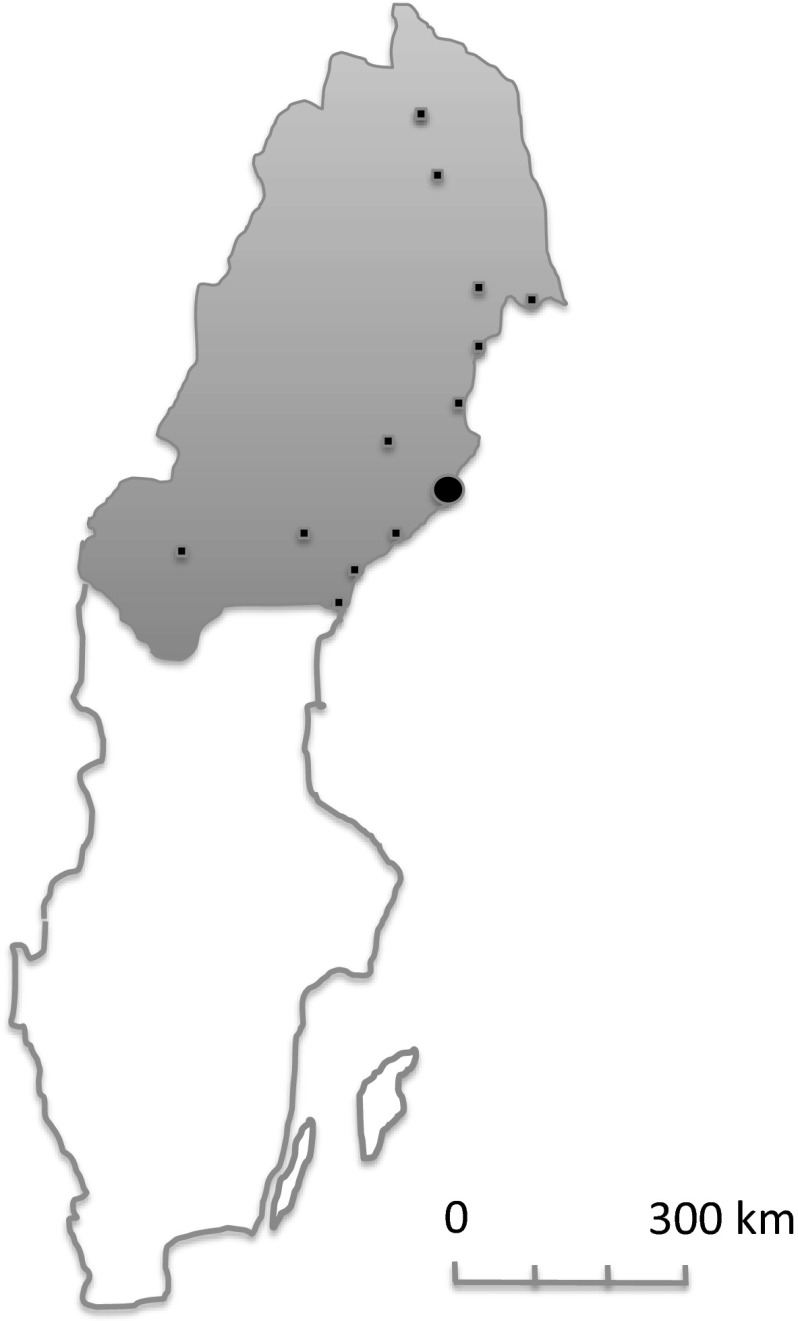
Northern Sweden Health Care Region. Median population 1995–2012 898 696 (scb.se). Cancer Prevention Clinic, Umeå University Hospital (*black circle*). Local hospitals (*black square*, smaller than the *circle*)

All cancer risk assessments were centralized to the Cancer Prevention Clinic at Umeå University Hospital, whereas the performance of the colonoscopies were decentralized, as these colonoscopies were performed by physicians and surgeons at local hospitals in northern Sweden (Fig. 1). There were no formal requirements on the examiner’s competence (e.g., minimum number of colonoscopies/year or adenoma detection rates). Before all planned colonoscopies, the cancer prevention center mailed a reminder to the local hospital and to the patient. The readings of the pathology specimens were also decentralized to the local hospitals.

The individuals in the registry were classified into six groups according to their risk for CRC (Table 1). The following two risk groups were excluded from the analyses: Individuals with a non-significantly increased cancer risk who received surveillance on psychological indication only (Group 1) and known carriers of adenomatous polyposis coli (APC) as these individuals have a separate standard for surveillance, which includes prophylactic surgery (Group 4).

**Table 1:** Classification of family history for estimation of life time risk for colorectal cancer (CRC), regional guidelines for start of surveillance and intervals

Risk group	Family history (FDR = first degree relative)	Start of surveillance	Intervals between colonoscopies
Familial colorectal cancer ( FCRC)			
l^a^	At least two relatives^b^ with CRC diagnosed over age 70	Individually	Individually
2 (2FDR)	2 FDR with CRC diagnosed under age 70	5–10 years before the age of first diagnosed CRC case in the family	5 years
3a (3FDR)	3 FDR with CRC diagnosed under age 70	5–10 years before the age of first diagnosed CRC case in the family	5 years
3b (Amsterdam-)	Fulfilling all Amsterdam criteria except one	5–10 years before the age of first diagnosed CRC case in the family	5 years
Hereditary colorectal cancer (HCRC)			
3c	Fulfilling Amsterdam criteria or MSI positive or MMR mutation regardless of family history	Age 25	2 years
4^a^ (FAP^c^)	Known APC carrier	Age 12	2 years

^a^Excluded from analysis

^b^First or second degree relatives

^c^Familial adenomatous polyposis (FAP) or Attenuated familial adenomatous polyposis (AFAP)

The current regional surveillance guideline (2009) in northern Sweden recommends an interval between colonoscopies of 2 years in Group 3c (HCRC) and 5 years for all other groups (FCRC) [7]. Before 2009, a three-year interval was sometimes used for individuals when the distinction between HCRC and FCRC was difficult to ascertain.

### Statistical analysis

The study’s main outcome measures looked at high-risk adenomas (HRA) or CRC and compliance with the surveillance program. HRA was defined as an adenoma with villous histology, ≥10 mm diameter, or with high grade dysplasia [8]. Findings were analyzed at the first surveillance and at the follow-up colonoscopies. To validate the findings of cancer, we linked all study subjects to the Regional Cancer Registry. The individual’s place of location was defined as a sparsely populated according to definitions by Swedish Association of Local Authorities and Regions [11].

To evaluate the effectiveness of the surveillance program in preventing cancer, we estimated the expected numbers of colorectal cancers for the study population without surveillance. These estimations were based on age-specific CRC incidence rates for the general population in Sweden [12] and on the relative risk for patients with HCRC or FCRC as proposed by Dowe-Edwin [8]. Dowe-Edwin’s data on relative risk are specific to age and risk group (family history) and are categorized as lowest, best, or highest estimate. To increase the reliability, we used two methods—A and B—to estimate the expected numbers of CRC in the study population.

Using method A, we calculated the annual expected numbers of CRC in the study population using age-specific general population rates multiplied with the relative risk according to age and family history, and then summed for the entire study period.

Using method B, an already developed model for cancer incidence simulation (Person Years, PYRS), estimated age, sex, and calendar year adjusted CRC incidence. PYRS has been described in detail elsewhere[13]. To compare observed versus expected cases of CRC, two tailed standard incidence ratios (SIR) with 95 % confidence intervals were calculated according to Byar’s formula.

To compare baseline characteristics between the risk groups, we performed independent *T* test or the Chi square test. To analyze differences in findings at colonoscopy between patients with different sex, age, and risk, we used binary logistic regression. The regression models were adjusted, when appropriate, for sex, age and risk. The analysis was performed in IBM^®^ Statistics SPSS^®^ for Mac, version 20 and 22.

The Regional Ethical Review Board in Umeå approved the study and all study subjects gave their informed consent to be included in the registry.

## Results

During the study period, 278 individuals from 118 different families were recorded in the registry and scheduled for 691 colonoscopies. All study subjects in Group 1 (very low risk, n = 10) and Group 4c (APC carriers, n = 7) were excluded from analysis. The remaining 261 study subjects overall compliance to the surveillance program was 90 % (597 of their planned 662 colonoscopies were performed). There were no significant differences in mean age (*p* = 0.23), sex (*p* = 0.18), risk group (*p* = 0.056) or place of location (*p* = 0.59) between non-compliant and compliant individuals. Overall was 36.4 % of the study population living in sparsely populated areas.

Of the performed examinations, 10 % (60/597) were not complete. Due to inadequate bowel cleaning 17 % (10/60) or failure to reach the caecum 83 % (50/60). Out of the incomplete examinations, 47 % (28/60) were later completed with a new colonoscopy or diagnostic imaging. There was no difference in the proportion of complete examinations between densely and sparsely populated areas (*p* = 0.44).

### First surveillance colonoscopies

The mean age for the first planned colonoscopy was 53 years, and more women (61 % 159/261) than men were registered. HCRC patients (Group 3c) were significantly younger compared to all FCRC patients (Groups 2, 3a, and 3b together) (50.7 vs. 55.4 years, *p* = 0.001) (Table 2). There was no significant difference in mean age between men and women (*p* = 0.20).

**Table 2:** Baseline characteristics for individuals in the registry for surveillance of familial (FCRC) or hereditary colorectal cancer (HCRC) in northern Sweden

Risk group	Families n (%)	Females n (%)	Males n (%)	Individuals n (%)	Mean age for planned first colonoscopy (range)
2 (2 FDR)	21 (19)	20 (74)	7 (26)	27 (100)	52.3 (32–72)
3a (3 FDR)	29 (27)	34 (55)	28 (45)	62 (100)	54.6 (34–75)
3b (Amsterdam-)	11 (10)	16 (55)	13 (45)	29 (100)	60.1 (39–79)
3c (HCRC)^a^	47 (44)	89 (63)	52 (37)	141 (100)	50.7^b^ (24–78)
Total	108 (100)	159 (61)	100 (39)	259^c^ (100)	52.8 (24–79)

Individuals in Groups 1 and 4 are not included

^a^Composition of HCRC group: 51.7 % MMR mutation carriers, 19.6 % Amsterdam positive but not mutation carriers, 28.7 % Amsterdam positive but not tested for MMR mutations

^b^HCRC patients (Group 3c) were significantly younger compared to all FCRC patients (Groups 2, 3a and 3b altogether) (*p* < 0.0001)

^c^Two study subjects excluded due to missing data on group or age

For the first surveillance colonoscopies, 80.6 % (191/237) were normal, 5.9 % (14/237) showed a HRA, and no cancers were found (Table 3). The first examination revealed more men than women (7.6 % (11/145) vs. 3.3 % (3/92), *p* = 0.008) with HRA. The probability of finding a HRA on the initial colonoscopy also increased with age (*p* < 0.0001), but was not associated to group (*p* = 0.79). Even if all FCRC groups (2, 3a, 3b) were considered as one group and compared to HCRC (3c), there was no significant difference in proportion of HRA (7.7 vs. 4.5 % *p* = 0.54). The youngest FCRC (Group 2, 3a, and 3b) patient with a HRA on the initial examination was 41 years old, whereas the youngest HCRC patient with HRA was 34 years old.

**Table 3:** Most advanced finding at first surveillance colonoscopy

Risk group	Normal	Metaplastic polyp	Simple adenoma	Multiple adenoma	High risk adenoma	Cancer	Total
2 (2 FDR)	19 (79.2)	2 (8.3)	1 (4.2)	1 (4.2)	1 (4.2)	0	24 (100)
3a (3 FDR)	40 (78.4)	1 (2)	5 (9.8)	0	5 (9.8)	0	51 (100)
3b (Amsterdam-)	22 (75.9)	2 (6.9)	1 (3.4)	2 (6.9)	2 (6.9)	0	29 (100)
3c (HCRC)	110 (82.7)	6 (4.5)	10 (7.5)	1 (0.8)	6 (4.5)	0	133 (100)
Total	191 (80.6)	11 (4.6)	17 (7.2)	4 (1.7)	14 (5.9)	0	237^a^ (100)

Values are number (%) of patients

^a^23 patients were never examined and one patient was excluded due to missing data on finding

### Follow-up time

The total follow-up time for the study subject’s risk of developing CRC was 1256 person years (time from first colonoscopy until last notification in the Local Cancer Registry, 1 September 2012). Individuals in Group 3c (HCRC) were followed for 796 person years For the risk of developing adenomas, the follow-up time was 760 person years, based on the 149 patients who were examined at least twice. The median time between their first and last colonoscopy was 5.1 years. The consistency between the recommended surveillance intervals by the genetic counselor and the regional guidelines for surveillance were over 80 % for all groups (Supplementary Table A).

### Follow-up surveillance colonoscopies

On the follow-up colonoscopies, one cancer and 12 high-risk adenomas were found and 281 of the 356 (78.9 %) examinations were normal (Table 4). All 13 patients with HRA or CRC had their follow-up colonoscopies within 4 months from their planned date according to their surveillance interval. However, in 3 cases, the previous colonoscopy was not complete.

**Table 4:** Most advanced finding at all follow up colonoscopies

Risk group	Normal	Metaplastic polyp	Simple adenoma	Multiple adenoma	High risk adenoma	Cancer	Total
2 (2 FDR)	12 (92.3)	1 (7.7)	0	0	0	0	13 (100)
3a (3 FDR)	36 (81.8)	3 (6.8)	4 (9.1)	0	1 (2.3)	0	44 (100)
3b (Amsterdam-)	28 (73.7)	4 (10.5)	3 (7.9)	1 (2.6)	2 (5.3)	0	38 (100)
3c (HCRC)	205 (78.5)	15 (5.7)	28 (10.7)	3 (1.1)	9 (3.4)	1 (0.4)	261 (100)
Total	281 (78.9)	23 (6.5)	35 (9.8)	4 (1.1)	12 (3.4)	1 (0.3)	356^a^ (100)

Values are number (%) of examinations

^a^Three examinations were excluded due to missing data on finding

The risk of finding a cancer or a HRA on follow-up was increased with the patient’s age (*p* < 0.0001) but was not associated to risk group (*p* = 0.94) or sex (*p* = 0.89). The youngest HCRC patient with HRA was 40 years old and the youngest FCRC patient was 50 years old. Although no individual in Group 2 (2 FDR with CRC) developed adenomas, it was not a statistically significant result. When all FCRC groups (2, 3a, and 3b) were compared as one group to HCRC (3c), there was still no significant difference in proportion of HRA or CRC (3.1 vs. 3.4 %, *p* = 0.67). The patient’s follow-up findings at colonoscopy were compared to the initial findings (Table 5). Adenomas (simple, multiple, high risk, or cancer) at the initial examination and increased age at follow-up were associated with adenomas at the follow-up (*p* = 0.007 and *p* = 0.003, respectively). However, among the seven FCRC patients >60 years old and without adenomas at the initial colonoscopy, none had developed adenomas at the follow-up.

**Table 5:** Relationship between patients’ most advanced finding on first colonoscopy and on any follow-up colonoscopy

Most advanced finding at first colonoscopy	Most advanced finding on any follow up colonoscopy					
	Normal	Metaplastic polyp	Simple adenoma	Multiple adenoma	High risk adenoma	Cancer
Normal (n = 116)	76 (65.5)	13 (11.2)	16 (13.8)	2 (17.2)	8 (6.9)	1 (0.86)
Metaplastic polyp (n = 7)	5 (71.4)	1 (14.3)	1 (14.3)	0	0	0
Simple adenoma (n = 8)	4 (50)	1 (12.5)	3 (37.5)	0	0	0
Multiple adenoma (n = 3)	1 (33.3)	0	0	1 (33.3)	1 (33.3)	0
High risk Adenoma (n = 9)	2 (22.2)	0	5 (55.6)	1 (11.1)	1 (11.1)	0
Total (n = 143^a^)	88 (61.5)	15 (10.5)	25 (17.5)	4 (2.8)	10 (7.0)^b^	1 (0.7)

Values are number of patients (%)

^a^Three patients were excluded due to missing data

^b^One patient had high risk adenomas on two follow-up colonoscopies, another patient had high risk adenoma on one follow u on cancer. Hence, there were 12 follow-up examinations (see Table 4)

No CRC associated deaths were reported, but seven individuals died of other causes during the study period. Two patients in group 3c who had very large HRA on their first colonoscopy and the patient with CRC needed surgical intervention.

The patient who developed CRC was a 70 year-old patient in Group 3c (HCRC) who fulfilled the Amsterdam criteria but was not tested for microsatellite instability or HNPCC genes. The cancer was diagnosed after diagnostic and treatment difficulties of a suspected earlier found HRA.

### Expected numbers of CRC

Without surveillance, the best estimate for expected numbers of CRC in the study population would range from 9.5 to 10.5 cases during the study period, depending on the statistical method used (Method A or B). The standardized incidence ratios (SIR), observed versus expected cases of CRC, based on the best estimates are between 0.10 (CI 95 % 0.0012–0.5299) and 0.11 (CI 95 % 0.0014–0.5857) (Table 6). This indicates a possible significant reduction in CRC due to surveillance.

**Table 6:** Estimate of expected number of cancers and observed number of cancers during the study period, including total standardized incidence ratio (SIR), observed versus expected (O/E)

Group	Estimated number of cancers						Observed number of cancers
	Method A			Method B (PYRS)			
	Lowest estimate	Best estimate	Highest estimate	Lowest estimate	Best estimate	Highest estimate	
2 (2 FDR)	0.1	0.2	0.3	0.1	0.2	0.3	0
3a (3 FDR)	0.4	1.0	1.8	0.4	1.0	1.8	0
3b (Amsterdam)^a^	0.7	1.5	2.7	0.7	1.6	3.0	0
3c (HCRC)	3.6	7.9	11	3.1	6.7	9.2	l
Total	4.9	10.5	16	4.3	9.5	14.2	l
Total SIR O/E (CI 95 %)	0.20 (0.0027–1.135)	0.10 (0.0012–0.5299)	0.06 (0.0008–0.3477)	0.23 (0.0030–1.294)	0.11 (0.0014–0.5857)	0.07 (0.0009–0.3918)	

^a^Group 3b (Amsterdam) was assessed as having a relative risk for CRC corresponding to patients in group 22 (3 FDR)

If the studied population would have had the same risk for CRC as the general population in Sweden, the expected numbers of CRC are approximately 0.8.

## Discussion

### Main findings

This study demonstrated that colonoscopic surveillance with high patient compliance prevented hereditary and familial CRC. In our study, only one of the 237 individuals developed CRC while under surveillance. The decentralized method for colonoscopy surveillance under the guidance of the cancer prevention clinic might have improved compliance.

### Prevention of colorectal cancer

In our study, the proportion of CRC in families with HCRC was only 0.7 % (1/134), or an incidence rate of one case in 796 years of follow-up. This incidence rate is comparable to a population without increased familial risk for CRC[12]. Three previous controlled trials of colonoscopic surveillance in Lynch syndrome, all comparing the outcome between unscreened and screened patients, report a proportion of CRC ranging from 3.5 to 10.9 % in the screened group [4, 14, 15]. These higher numbers can partly be explained by a longer surveillance interval in one of the earlier trials[4], but two recent studies use the same intervals as in our program (i.e., 2 years) [14, 15]. However, comparisons between controlled and prospective studies should be made with caution, as many of these studies use different definitions of HCRC and Lynch syndrome.

Only a few prospective studies present results of surveillance of both HCRC and FCRC.

A large study by Dowe-Edwin et al. (2005) includes patients both with HCRC (surveillance with 2-year intervals) and FCRC (surveillance with 5-year intervals) and reports a CRC incidence in the same order of magnitude as in our study, 1/1200 person years versus 11/11,000 person years [8]. Their recommended start of surveillance was 25 years for both HCRC and FCRC, with a lower mean age at first examination than in our study (41 vs. 53 years). However, the earlier start of surveillance of the FCRC group does not seem to have increased the efficiency in preventing CRC.

A recent prospective multicenter study by Mesher et al. reports an incidence of CRC of 1.14 per 1000 person-years, but the surveillance strategy varied at the different participating centers (1–5 year intervals) [16].

In summary, the CRC preventive effect in our study was equal to or better than the effect shown in other studies.

An important factor for preventing CRC is the patient’s compliance to the surveillance program. In our study, the patient compliance was very high (90 %), compared to participation rates reported (10–39 %) from screening programs in the general population [17, 18]. We have not found information on compliance in previous reports on surveillance of FCRC or HCRC. Participation rates for cancer screening are assumed to be higher in rural areas and among females [18, 19]. In our study there was no difference in compliance between sparsely and densely populated areas according to Swedish definitions. However compared with most other countries, almost the entire northern Sweden could be considered a sparsely populated area. Consequently, it is difficult to determine if the high compliance in our study was due to the decentralized organization of the surveillance, or by an overall rural setting and a predominance of female study subjects. This may affect the possibility to reproduce high compliance with decentralized surveillance in another setting.

Another factor affecting CRC prevention might be the quality of the colonoscopies. Our reported rate of 10 % incomplete colonoscopies is high compared to international quality targets for CRC screening [6, 20]. The reason may be the lack of nationwide quality assurance guidelines for colonoscopies in Sweden. The slightly lower quality of the colonoscopies in our study has nevertheless not resulted in a low CRC preventive effect.

However, without any kind of modeling is it hard to determine how important compliance and quality of colonoscopies are for CRC prevention compared to other factors as start of surveillance and intervals.

### Start of surveillance and intervals

An accelerated adenoma-carcinoma pathway is often used to explain the increased risk for patients with a family history of CRC [21]. Theoretically, the speed of the pathway decides at what age surveillance should begin and how often a patient should be re-examined to detect new pre-malignant adenomas. Hence, evaluation of the findings at the initial colonoscopy may optimize the timing for the start of surveillance, whereas the length of intervals is best determined by the findings gathered from follow-up examinations.

In this study, more men than women had HRA at the initial colonoscopy, whereas there were no gender differences at the follow-up examination. The difference at the first examination may have been caused by an increased background risk because men typically have more HRA and CRC than women [22, 23].

At the first examination, individuals with FCRC had a mean age of 55.4 years and 7.7 % had HRA. In a population without increased risk for CRC, the rate of HRA has been reported to be 3.8 % for all patients <65 years or 5.7 % for patients 40–49 years old [23, 24]. The higher proportion of HRAs in our population is expected because of their overall increased risk for CRC, but it is not clear at what age HRAs start to develop in individuals with FCRC. Our findings are consistent with other studies of FCRC, reporting a sharp increase in the proportion of HRA on the initial examinations around age 50 [8]. Consequently, starting surveillance for FCRC between 40 and 50 years or 5–10 years before the first case of CRC in the family seems reasonable. However, in this study, albeit with low subgroup numbers, patients over 60 years old with FCRC and no adenomas on the initial colonoscopy seem to have a very low risk of developing any adenomas by the follow-up. If confirmed in other larger studies, a single colonoscopy could be a future strategy for FCRC surveillance [3].

On the follow-up colonoscopies, there was no difference between HCRC and FCRC in the rate of HRA and CRC (3.1 vs. 3.4 %). Earlier studies conclude patients with HCRC have an accelerated adenoma-carcinoma pathway and new HRA can develop from a clean colon in a few years [4, 8, 21, 25]. Our findings suggests that colonoscopic surveillance with a two-year interval in HCRC and a five-year interval in FCRC equalized the risk for development for HRA between HCRC and FCRC and reduced the risk of CRC to that of the general population (i.e., average risk). However, if all CRC is to be prevented, a shorter interval in HCRC might be necessary, a recommendation found in the British guidelines for Lynch syndrome [3].

More frequent colonoscopies with detection and polypectomy of simple adenomas before progression may prevent development of HRA. To prevent all HRA would require an intensive surveillance regimen resulting in high health care costs and possible discomfort for the patients. But what is the optimal HRA detection rate in a surveillance program? From a health economic perspective, it might be sufficient to detect any HRA early enough to allow endoscopic polypectomi instead of surgery. In our study, 7.6 % (2/26) of patients with HRA required surgical intervention, a low percentage compared to other studies [26, 27].

### Limitations

The major limitation of this study is the lack of a control group, which necessitates an estimate of the expected cases of CRC without surveillance. However, with the knowledge we have today, it would be considered ethically problematic to randomize patients with an increased risk of CRC to non-surveillance. Hence, the best available option to optimize surveillance in the future is prospective studies that focus on different on-going surveillance programs. Another weakness is the limited number of patients in the different FCRC risk groups. Valid subgroup analysis becomes difficult, especially for low risk patients where the beneficial effects of surveillance might be low.

The median follow-up time of approximately 5 years may also be too short; many patients may have not been followed long enough to develop a CRC.

### Strengths of the study

The main strength of the study is the quality of the follow-up using Sweden’s unique personal identification number and the link of all patients to the Local Cancer Registry. The Cancer Registry started in 1958, and its validity on cancer data in Sweden is over 95 %, which makes the possibility of further unreported cases of CRC low [28]. The cancer history in the families are validated through the Cancer Registry and through saved medical records, providing robust information of the exact numbers and age at diagnosis of cancer among family members. Our study is also one of the few studies to report compliance with surveillance [29].

## Conclusions

Our study provides a reasonably safe strategy for surveillance of FCRC and HCRC with high patient compliance in a sparsely populated area by using decentralized colonoscopies. However, health economic analyses and modeling are needed to find the most cost effective way to prevent cancer development in individuals with a family history of CRC. These future studies of surveillance programs should include patient compliance as an important factor and not only focus on start of surveillance and the lengths of the intervals.

## Electronic supplementary material

Below is the link to the electronic supplementary material.

Supplementary material 1 (DOCX 374 kb)
