# Supplementary figures and images for: Decentralized colonoscopic surveillance with high patient compliance prevents hereditary and familial colorectal cancer
Source: Fam Cancer. 2016 Mar 2;15(4):543–51. doi: 10.1007/s10689-016-9867-7 (PMC5010828; doi:10.1007/s10689-016-9867-7)

## Supplementary Table A


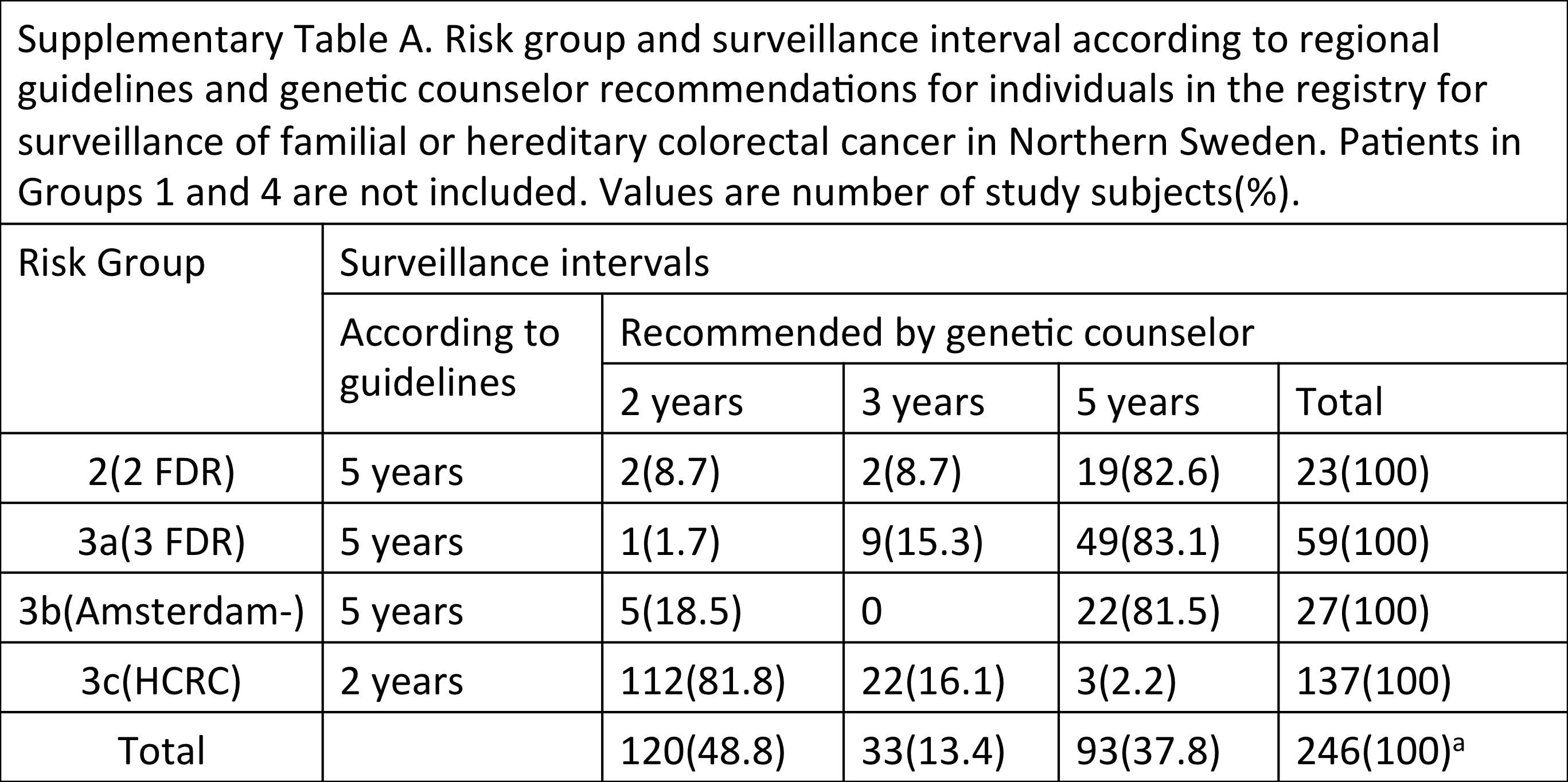


a 15 study subjects excluded due to missing data on group or interval.

Supplement: Supplementary file 1 — Supplementary material 1 (DOCX 374 kb) [file 10689_2016_9867_MOESM1_ESM.docx]
